# Seasonal Shifts of Morphological Traits and Dietary of *Mactra veneriformis* (Bivalvia: Mactridae) Populations in the Northern Yellow River Delta’s Intertidal Zone

**DOI:** 10.3390/biology14020176

**Published:** 2025-02-10

**Authors:** Shuangfeng Xu, Ang Li, Ling Zhu, Biao Wu, Lulei Liu, Minghui Jiao, Jiaqi Li, Suyan Xue, Yuze Mao

**Affiliations:** 1 College of Fisheries and Life Science, Shanghai Ocean University, Shanghai 201306, China; 17616155981@139.com; 2 State Key Laboratory of Mariculture Biobreeding and Sustainable Goods, Yellow Sea Fisheries Research Institute, Chinese Academy of Fishery Sciences, Qingdao 266071, China; lia@ysfri.ac.cn (A.L.); wubiao@ysfri.ac.cn (B.W.); liull@ysfri.ac.cn (L.L.); jiao_ming_hui2022@163.com (M.J.); lijq@ysfri.ac.cn (J.L.); xuesy@ysfri.ac.cn (S.X.); maoyz@ysfri.ac.cn (Y.M.); 3 Laboratory for Marine Ecology and Environmental Science, Qingdao Marine Science and Technology Center, Qingdao 266237, China

**Keywords:** seasonal variation, geometric morphometrics, dietary composition, high-throughput sequencing

## Abstract

In order to examine the seasonal variations in the morphological characteristics and diet of *Mactra veneriformis* in the Northern Yellow River Delta’s intertidal zone and provide a scientific basis for its resource conservation and population restoration, tested clams were collected in four consecutive seasons from summer of 2022 to spring of 2023. Morphological traits were measured, and the DNA of the stomach contents was analyzed using high-throughput sequencing. The tidal differences and seasonal variations in the northern habitat of the Yellow River Estuary significantly affect the morphological characteristics and growth of *M. veneriformis*. Among the four seasons, significant differences in the morphological characteristics of *M. veneriformis* were observed between the middle-tide and low-tide zones (*p* < 0.05). In both middle-tide and low-tide zones, the morphological characteristics and body wet weight of *M. veneriformis* in winter were significantly higher than those in other seasons (*p* < 0.05). Moreover, the morphological characteristics of *M. veneriformis* were extremely significantly influenced by the interaction between tide and season (two-way interaction, *p* < 0.001). In all seasons, *M. veneriformis* in the middle- and low-tide zones exhibited positive allometric growth. While there was no significant difference in the stomach content between the spring and summer samples in the same tidal zone (*p* > 0.05), there was a significant difference between the middle- and low-tide samples in winter (*p* < 0.05). This suggests that seasonal variations, rather than tidal differences, had a larger impact on the diet of *M. veneriformis* in the northern Yellow River estuary and that feeding differences may be related to changes in environmental factors, such as temperature. The findings of this study provide initial insights into the feeding ecology of *M. veneriformis* and offer a scientific foundation for the conservation and management of its resources.

## Simple Summary

Morphological traits and dietary habits are important aspects of shellfish biology and ecology, forming the foundation for fishery resource development and management. This study investigated *Mactra veneriformis* in the intertidal zone of the northern Yellow River Delta. Morphological traits (shell length, shell height, shell width, and body mass) were measured, and stomach DNA was analyzed using high-throughput sequencing to determine the dietary composition. The results showed that tidal differences in the northern Yellow River estuary had minimal influence on the quantitative traits and growth of *M. veneriformis*, and the dietary differences were mostly influenced by seasonal variations.

## 1. Introduction

Coastal wetlands have high productivity and rich biodiversity and are important components of “blue carbon” ecosystems [1]. The Yellow River Delta, located on the south shore of Bohai Bay and the west shore of Laizhou Bay, is the most preserved, extensive, and fastest-growing wetland ecosystem in China’s warm-temperature zone. The mudflats are silty and sandy, with flat terrain, nutrient-rich water, and abundant phytoplankton, resulting in a major shellfish production area in Shandong Province [2]. *Mactra veneriformis* W. Wood, 1828 is the most important economic shellfish in the Yellow River Delta mudflats, boasting the highest yield and production value [3].

*M. veneriformis* is mainly distributed in the middle and low intertidal zones of the Yellow River Delta at an extremely high habitat density and biomass. As a filter-feeding bivalve, *M. veneriformis* primarily feeds on phytoplankton and organic detritus in the water column [4], and they play an important role in the biological coupling between the pelagic and benthic environments through processes such as feeding and biodeposition. Many studies have been conducted on their resource reserves, distribution areas, and community structure [2,5,6,7], but research on their population morphological characteristics and feeding habits is needed.

Costa et al. [8] stated that the contribution of local adaptation to the morphological differentiation of clam populations is still poorly studied. The intertidal zone is located in a sensitive zone of land–sea interaction and is simultaneously influenced by two major ecosystems, marine and terrestrial. The regular rise and fall of the tides result in differing periods of exposure to air and submersion for the different tidal zones, leading to significant differences in salinity, temperature, and other ecological factors [9]. Morphological variation in shellfish represents an ecological adaptation. Shellfish exhibit strong phenotypic environmental plasticity, and their morphological characteristics are not only influenced by genetics but are also influenced by the habitat [10]. In most cases, seasonal variations in the morphological characteristics of shells, such as shell length, width, height, and other morphological features [11], are closely related to environmental factors (temperature, salinity, food resources, and tides) and growth stages [12,13]. For example, Sousa et al. [14] found significant differences in the morphological characteristics of *Corbicula fluminea* (O. F. Müller, 1774) in two different estuaries in Portugal, and Galtsoff et al. [15] found significant differences in the length/width ratio of the long axis of the intertidal and subtidal *Crassostrea virginica* (Gmelin, 1791). In the study of *Crassostrea macroura* (G. B. Sowerby II, 1842), it was found that the density of scallop shells was higher at higher temperatures, while the shells of scallops were longer and narrower at higher salinities. When the food supply is insufficient, scallop shells become longer to increase predation area [16]. In addition, samples collected in different seasons may be at different growth stages, which can also lead to morphological differences. For example, samples collected in spring may be in the early stages of growth, while samples collected in autumn may have entered the mature stage and their morphological characteristics may differ. Therefore, *M. veneriformis* can adapt to different ecological niches by changing its morphological characteristics. Thus, *M. veneriformis* may adapt to different ecological niches by altering their morphological characteristics.

Dietary analysis is an important part of basic ecology, which can provide the food composition of aquatic organisms and a scientific basis for the development of strategies to enhance and conserve aquatic biological resources [17,18]. Currently, microscopy [19], stable isotope analysis [20], and fatty acid analysis [21] have been used to study the feeding habits of aquatic organisms, but there are certain limitations of each method. Microscopy is not suitable for assessing long-term ingestion and easily digestible food species or species at a low abundance [22]. In contrast, fatty acid markers and stable isotope analysis can characterize a broad range of food sources but have limited use for accurately determining detailed food composition [23,24]. With the advancement of molecular biology technology, high-throughput sequencing is being used for the identification of species within the stomach contents. This technique requires fewer samples, offers high sensitivity and accuracy, and can more effectively reveal the feeding habits and preferences of aquatic organisms. Consequently, this method provides more comprehensive and reliable information on food composition [25]. Currently, this technique has been used to study the feeding habits of a variety of aquatic organisms, such as *Crassostrea gigas* (Thunberg, 1793) [26], *Ophisternon candidum* (Mees, 1962) [27], *Sardina pilchardus* (Walbaum, 1792) [28], and *Heliocidaris crassispina* (Agassiz, 1864) [29].

Recently, shellfish resource harvesting in the mudflats of the Yellow River Delta has been increasing, and the issue of unsustainable shellfish production patterns has become increasingly prominent. As previous studies have not focused on the seasonal variations in the morphological characteristics and feeding patterns of *M. veneriformis*, we attempted to explore this aspect. In this study, the main economically important shellfish, *M. veneriformis*, was selected as the focus of this study, and its morphological characteristics were analyzed using allometric growth analysis. Additionally, its diet was analyzed using high-throughput sequencing technology to understand the differences across different seasons. This study aims to provide fundamental data on the morphology and feeding ecology of shellfish. By examining the seasonal changes in shellfish morphology and diet, we can gain a deeper understanding of their ecological requirements and thereby develop more science-based conservation measures. This research helps to better protect the genetic resources of shellfish and prevent species resource decline caused by overfishing or environmental degradation.

## 2. Materials and Methods

### 2.1. Sample Collection and Processing

**Figure 1:**
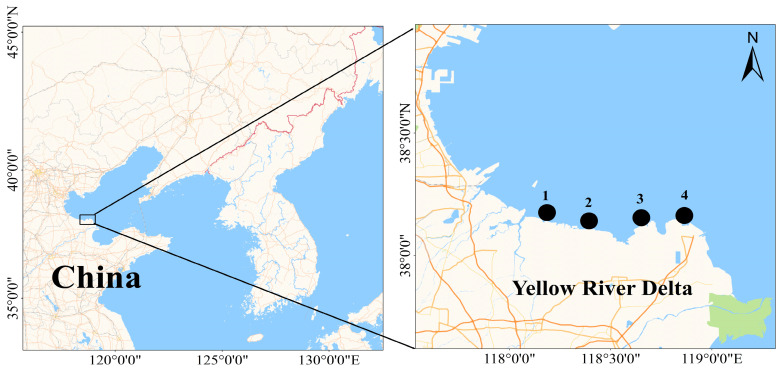
Schematic diagram of the sampling area of the intertidal shellfish resources survey in the northern Yellow River Delta.

The four sampling areas (1–4) of the northern intertidal zone of the Yellow River Delta were selected as the study area (Figure 1), and *M. veneriformis* were randomly collected from the estuary in August 2022 (summer), October 2022 (fall), February 2023 (winter), and May 2023 (spring). Three stations were set up in each section according to the high-, middle-, and low-tide zones, and three sample squares were set up in each station. Surveys are conducted during high tide ebb. The seawater temperature was measured using a multiparameter water quality analyzer (AquaTroll 400, In-Situ Inc., Fort Collins, CO, USA). During the sampling process, no shellfish samples were collected in the high-tide zone. Thus, *M. veneriformis* samples were analyzed in the middle- and low-tide zones. According to the season, samples in the low-tide zone were termed NLSp, NLSu, NLAu, and NLWi, and samples in the middle-tide zone were termed NMSp, NMSu, NMAu, and NMWi for spring, summer, autumn, and winter, respectively. Samples were brought back to the laboratory for species identification by specialized personnel. One hundred samples were randomly selected from each sample group, and their morphological traits (shell length, shell height, and shell width) and body mass were measured. The shell length, shell width, and shell height of *M. veneriformis* were measured using digital calipers (accuracy: 0.01 mm). The body wet weight of *M. veneriformis* was measured using an electronic balance (accuracy: 0.01 g). Four *M. veneriformis* individuals were randomly dissected from each sample group to obtain the stomach contents, and they were stored in liquid nitrogen and brought back to the laboratory for further processing. It is important to note that our study was limited by a relatively small sample size, with only four specimens per sample group. This limitation may affect the robustness of our extrapolations and should be considered when interpreting the results.

### 2.2. DNA Extraction

The stomach contents were washed with phosphate buffer (pH 7.2–7.6) and homogenized in the laboratory. After homogenization, they were quantified, and the total DNA was extracted using the Marine DNA Extraction Kit (Beijing Tiangen, Beijing, China) according to the protocol. The sample DNA was amplified by polymerase chain reaction (PCR) using the 23S ribosomal RNA (rRNA) primers p23SrV_f1 (5′-GGA CAG AAA GAC CCT ATG AA-3′) and p23SrV_r1 (5′-TCA GCC TGT TAT CCC TAG AG-3′) [30], and the PCR-amplified products were detected and purified by 2% agarose gel electrophoresis. Then, sequencing was conducted using the Illumina MiSeq sequencing platform (Illumina, San Diego, CA, USA) by Shanghai Personal Biotechnology Co. (Shanghai, China).

### 2.3. Sequencing Data Processing

The sequencing data were preliminarily screened, and chimeras were removed. The sequences were grouped into operational taxonomic units (OTUs) based on 97% sequence similarity using QIIME software (2019.4, https://library.qiime2.org, accessed on 12 June 2024), and the representative sequences within the OTUs were selected for species annotation analysis based on the National Center for Biotechnology Information database.

### 2.4. Data Analysis

We initially calculated the morphological characteristic index of four cornered clams in different seasons in the same tidal zone. Subsequently, we compared these indices in different seasons within the same tidal zone and in different tidal zones within the same season. This method allowed us to carefully examine the index changes caused by seasons and tidal zones. Essentially, we used a two-way ANOVA analysis of variance to determine whether there were differences between different populations in different locations and seasons. The significance level was set at *p* < 0.05, and *p* < 0.01 was considered to be highly significant. The allometric growth was calculated as follows:

Allometric growth equation: W = a × L^b.^

where W represents the body wet weight; a is the condition factor; L is the shell length; and b is the allometric growth parameter. If b < 3, it indicates negative allometric growth; if b = 3, it indicates isometric growth; if b > 3, it indicates positive allometric growth [31,32]. The growth curves were divided into different tidal zones, and a *t*-test was conducted to determine whether the growth parameter was equal to 3. If the test was significant, the growth was considered to be allometric; if it was not significant, it was considered to be isometric.

The alpha diversity indices (Chao1 index, observed species, Shannon diversity index, Simpson diversity index, Pielou’s evenness, and Good’s coverage index) [33] were calculated, and the Kruskal–Wallis rank sum test was used to analyze differences between the groups. Dunn’s test was used as a post hoc test to verify the significance of the differences.

Beta diversity analyses of the community structure among the different groups of species were conducted at the genus level using Non-metric Multidimensional Scaling (NMDS) based on the Bray–Curtis distance algorithm. Stress was used to measure the strength of the NMDS analysis results, where Stress < 0.05 indicates a good representation, Stress < 0.1 indicates a good ranking, and Stress < 0.2 indicates interpretive significance. The analysis of similarities (ANOSIM) was used to test whether the differences in the community structure were significant, and the test statistic (*R*) takes a value between −1 and 1. A value less than 0 means that the difference within the group is greater than the difference between groups, and a value greater than 0 means that the difference between groups is greater than the difference within groups [34]. All the sample groups were subjected to hierarchical cluster analysis, and species with significant differences between the groups were identified using Linear Discriminant Analysis of Effect Size (LEfSe) with thresholds of LDA Score > 2 and *p* < 0.05 [35]. All the data analyses were conducted using R 3.6.2 [36].

## 3. Results

### 3.1. Quantitative Trait Parameter Statistics

**Figure 2:**
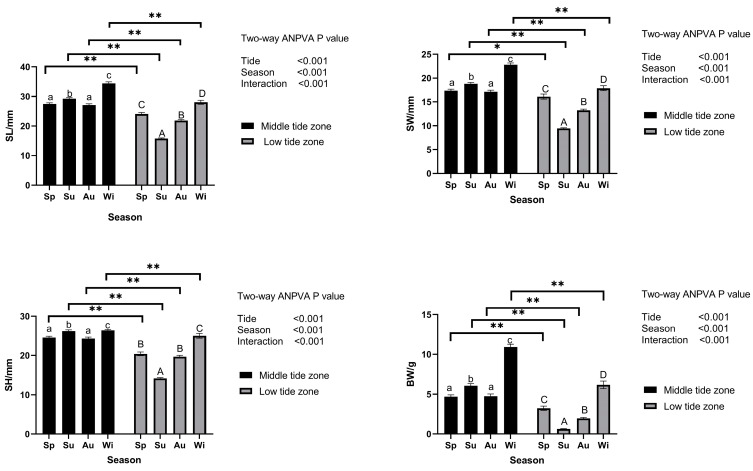
Two-factor ANOVA of morphological characteristics and body wet weight of *M. veneriformis*. The lowercase letters a, b, and c in the figure represent the differences between the four seasons in the mid tide zone, while the uppercase letters A, B, C, and D represent the differences between the four seasons in the low tide zone. *p* < 0.05 *, *p* < 0.01 **.

A two-way ANOVA was conducted on the morphological traits and body wet weight of *M. veneriformis* across different tide zones and seasons, with the results shown in Figure 2. The analysis indicated that in all four seasons, the morphological characteristics and body wet weight of *M. veneriformis* in the middle tide zone were significantly higher than those in the low-tide zone (*p* < 0.05). In both middle and low tide zones, the morphological characteristics and body wet weight of *M. veneriformis* in winter were significantly higher than those in other seasons (*p* < 0.05). Moreover, the morphological characteristics of *M. veneriformis* were extremely significantly influenced by the interaction between tide zone and season (two factors, *p* < 0.001). Seasonal variations in temperature are shown in Table 1, and only the fall and winter seasons were significantly different from the spring and summer seasons (*p* < 0.05).

### 3.2. Allometric Growth Analysis

The allometric growth equations of the shell length and body wet weight of *M. veneriformis* in the different tidal zones across the four seasons are shown in Table 2.

After significance analysis, all the allometric growth parameters in the fitted curves showed highly significant differences (*p* < 0.01). The b-values of the allometric growth parameters for shell lengths in the spring tidal zone were 3.703 and 3.179, respectively; in the summer tidal zone, they were 3.702 and 3.312, respectively; in the fall tidal zone, they were 3.738 and 3.321, respectively; and in the winter tidal zone, they were 3.670 and 3.422, respectively. The *t*-test shows that there is a significant difference in the wet weight of living organisms with an allometric growth index b > 3 and b = 3 in the four seasons of the mid low-tide zone (*p* < 0.05), i.e., the middle and low tide zones showed positive allometric growth in all seasons, and the growth rate of shell length was less than that of body wet weight.

### 3.3. Operational Taxonomic Unit Division and Alpha Diversity Index

A total of 1,201,441 high-quality sequence fragments were obtained from all the samples. The lowest sequence number was 29,161 and the highest sequence number was 72,612. A total of 1368 OTUs were obtained by clustering the sequences with 97% concordance, and the results of the OTU delineation and identification are shown in Table 3. The alpha diversity, or within-habitat diversity, was characterized by the Chao1 and observed species indices for richness, the Shannon and Simpson indices for diversity, Pielou’s evenness index [37] for evenness, and Good’s coverage index [38] for coverage.

**Figure 3:**
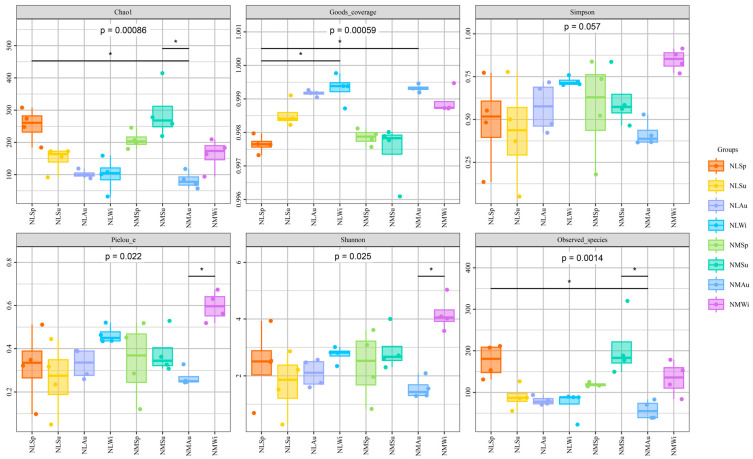
Analysis of overall alpha diversity. *p* < 0.05 *.

There were no significant differences in the Simpson index for all the groups (Figure 3). The NLSp and NLWi groups were significantly different for Good’s coverage (*p* < 0.05), and the NMSu and NMAu groups had significantly different Chao1 indexes and numbers of observed species (*p* < 0.05). Pielou’s evenness and Shannon index were significantly different for the NMAu and NMWi groups (*p* < 0.05), while there was no significant difference between the indices in the other groups (*p* > 0.05).

### 3.4. Analysis of Species Composition

**Figure 4:**
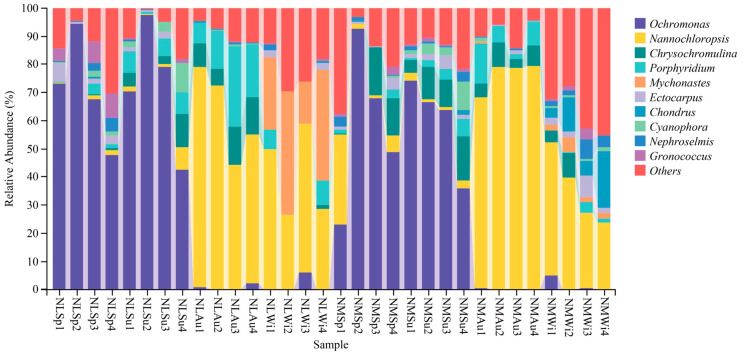
Species composition of eukaryotes.

Analysis of the taxonomic composition at the genus level showed that *Ochromonas* was abundant in both the NLSp and NLSu groups, with a mean of 70.82% and 72.46%, respectively, while other species were less abundant. *Nannochloropsis* was the most abundant in both the NLAu and NLWi groups, with a mean of 61.94% and 39.56%, respectively. *Porphyridium* and *Chrysochromulina* had mean contents of 17.04% and 10.33%, respectively, in the NLAu group, while Mychonastes had a mean content of 30.90% in the NLWi group (Figure 4). The trends of the groups in the middle and low tidal zones were generally consistent in the same seasons. In the NMSp and NMSu groups, *Ochromonas* had the highest mean content, at 58.25% and 60.14%, respectively, while the other species were less abundant. In the NMAu and NMWi groups, *Nannochloropsis* had the highest mean content, at 76.39% and 34.38%, respectively, with the other species in the NMAu group being less abundant.

### 3.5. Analysis of the Differences in Species Composition

**Figure 5:**
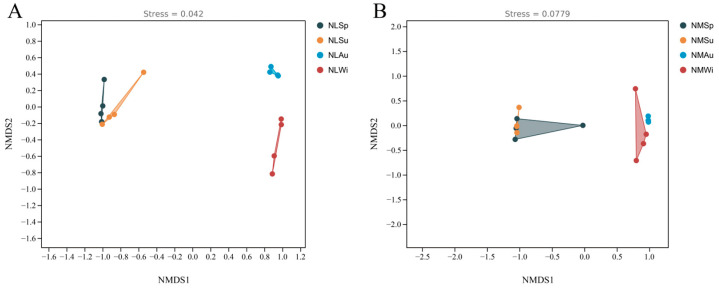
Non-metric multidimensional scaling in different seasons: (**A**) low-tide zone; (**B**) middle-tide zone.

NMDS is a non-metric multidimensional scaling method, using high-throughput sequencing results from samples collected in different seasons from mid- and low-tide zones. Through NMDS analysis based on the Bray–Curtis distance, it aims to explore the similarities and differences between different sample groups in different tide zones (low-tide zone and mid-tide zone) and display the distribution of different sample groups in the principal component space. The NMDS analysis results showed that in the low-tide zone, the NLSp and NLSu groups clustered together, while the NLAu and NLWi groups were clustered separately (Figure 5A). In the middle-tide zone, the NMSp and NMSu groups clustered together, while the NMAu and NMWi groups were relatively similar (Figure 5B). The ANOSIM analysis results indicated that in the low-tide zone, there were no significant differences between the NLSp and NLSu samples (*p* > 0.05), while significant differences were observed among the other samples (*p* < 0.05). In the middle-tide zone, there were no significant differences between the NMSp and NMSu samples (*p* > 0.05), but significant differences were observed among the other samples (*p* < 0.05).

**Figure 6:**
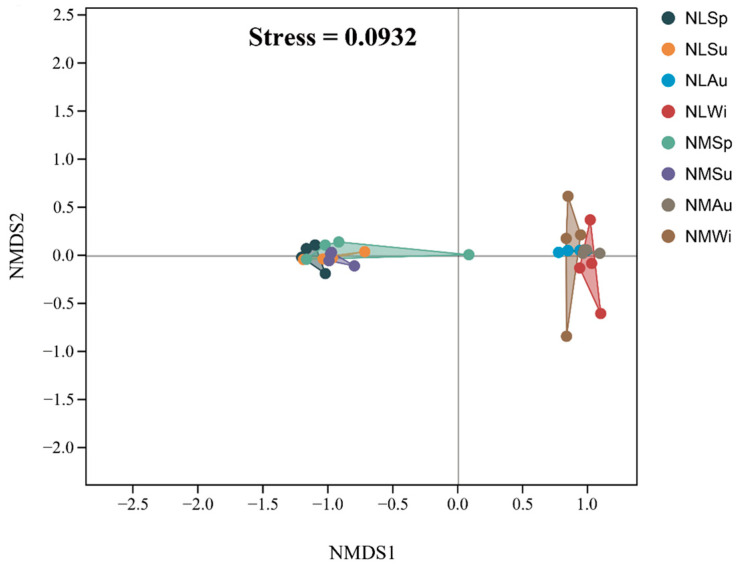
Analysis of overall beta diversity: non-metric multidimensional scaling.

Regarding the overall beta diversity analysis, the NMDS analysis showed that the spring and summer samples in the middle- and low-tide zones largely clustered together, while the fall and winter samples also clustered together (Figure 6). The Stress was 0.0932, indicating a good sorting result. Additionally, there were significant differences in the ANOSIM analysis in terms of the species composition (R = 0.72, *p* < 0.05), and the differences between groups were greater than those within groups. There were no significant differences (*p* > 0.05) between the samples in the same tidal zone in spring and summer. However, in winter, there was a significant difference (*p* < 0.05) between the samples from the middle- and low-tide zones.

**Figure 7:**
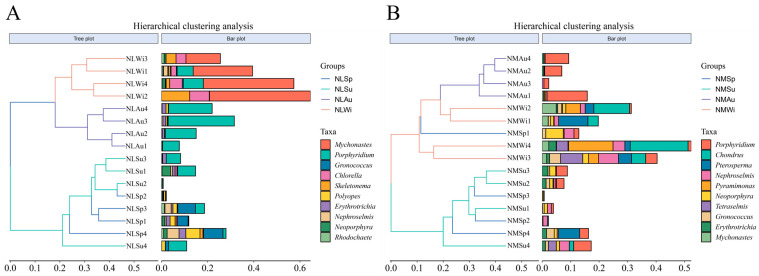
Hierarchical clustering analysis in different seasons: (**A**) low-tide zone; (**B**) middle-tide zone.

The clustering analysis results showed that the NLSp and NLSu groups clustered together in the low-tide zone, and the NLAu and NLWi groups clustered together (Figure 7A). In the middle tidal zone, the NMSp and NMSu groups clustered together, while the NMAu and NMWi groups clustered together (Figure 7B).

**Figure 8:**
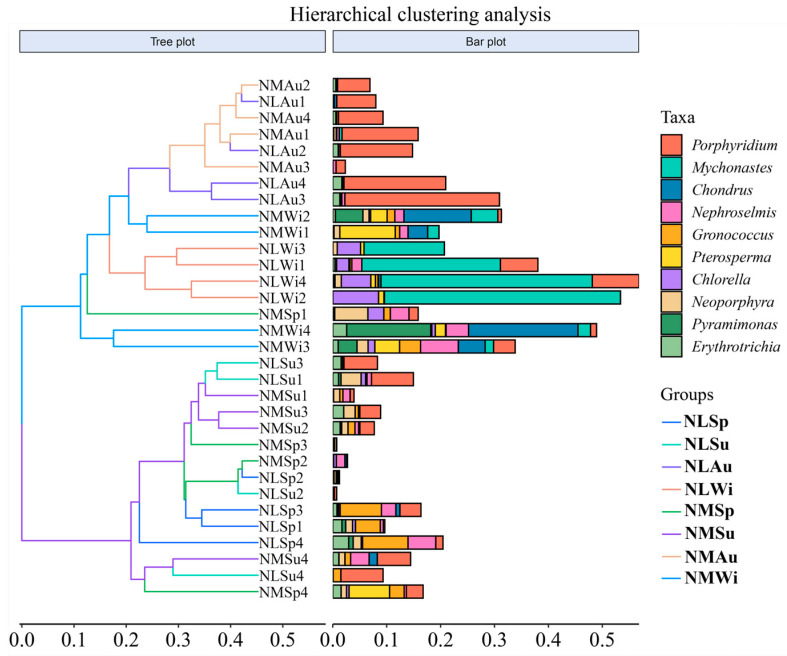
Overall hierarchical clustering analysis.

All the groups were pooled for the cluster analysis, and the overall results showed that there were essentially two clusters. The autumn and winter samples from the middle- and low-tide zones were clustered into one group, and the spring and summer samples from the middle- and low-tide zones were clustered into another group (Figure 8).

**Figure 9:**
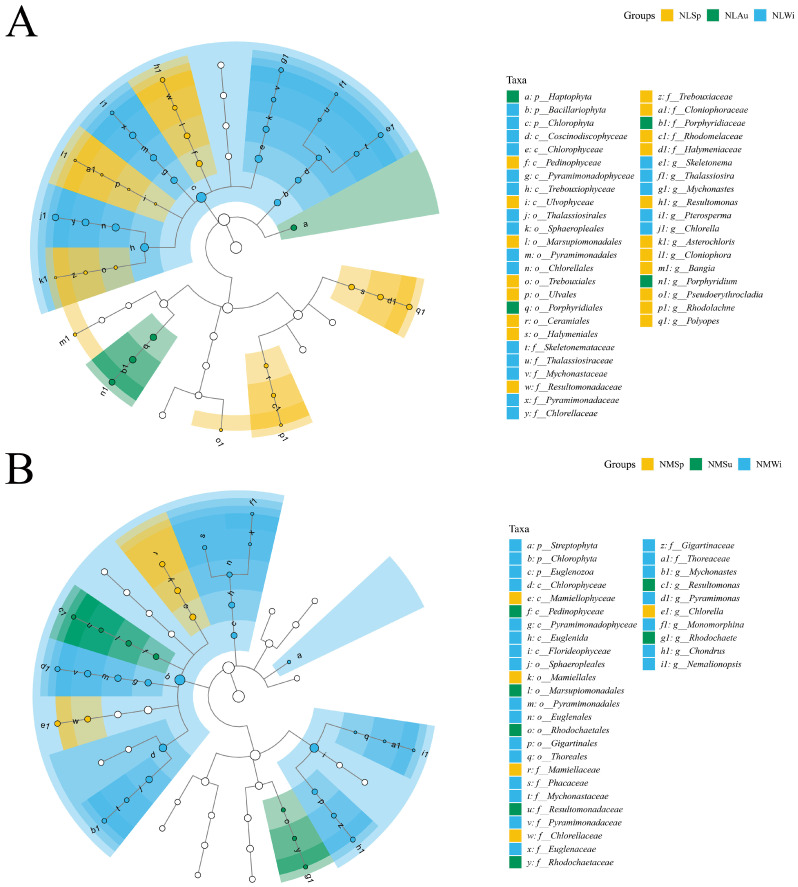
Linear discriminant analysis of the effect size in different seasons for the (**A**) low-tide zone and (**B**) middle-tide zone.

For the LEfSe analysis results, in the low-tide zone, there were 13 species with significant differences at the genus level (Figure 9A), namely Skeletonema, Thalassiosira, Mychonastes, Pterosperm, and Chlorella in the NLWi group, Porphyridium in the NLAu group, and Resultomonas, Asterochloris, Cloniophora, Bangia, Pseudoerythrocladia, Rhodolachne, and Polyopes in the NLSp group. In the middle-tide zone, there were eight species with significant differences at the genus level (Figure 9B), specifically Mychonastes, Pyramimonas, Monomorphina, Chondrus, and Nemalionopsis in the NMWi group, Resultomonas and Rhodochaete in the NMSu group, and Chlorella in the NMSp group.

**Figure 10:**
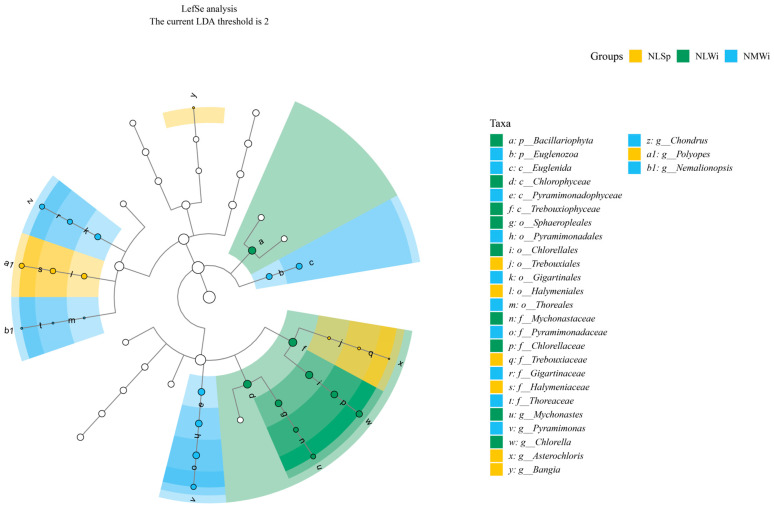
Overall linear discriminant analysis of the effect size.

For the overall LEfSe analysis, there were eight species with significant differences at the genus level (Figure 10); specifically, Asterochloris (Chlorophyta), Bangia (Rhodophyta), and Polyopes (Rhodophyta) in the NLSp group, Mychonastes (Chlorophyta) and Chlorella (Chlorophyta) in the NLWi group, and Pyramimonas (Chlorophyta), Chondrus (Rhodophyta), and Nemalionopsis (Rhodophyta) in the NMWi group. There were no species with significant differences in the other groups.

## 4. Discussion

### 4.1. Analysis of the Morphological Traits

In this study, in the mid tidal zone, the morphological traits of *M. veneriformis* in winter were significantly different from those in other seasons (*p* < 0.05), while no significant differences were observed in spring and autumn (*p* > 0.05). In the low-tide zone, the morphological traits of *M. veneriformis* in winter were significantly different from those in other seasons (*p* < 0.05), while shell height showed no significant differences in spring and autumn (*p* > 0.05). This indicates that seasonal changes have a significant impact on the morphological traits of *M. veneriformis*. Additionally, significant differences were observed in the morphological indices of *M. veneriformis* between the middle-tide and low-tide zones during the same season (*p* < 0.05). This may be due to the environmental variations in these zones, which lead to changes in shell morphology. It suggests that tidal variations in the northern habitats of the Yellow River Delta have an impact on the morphological indices of *M. veneriformis*. The two-way ANOVA indicated that the interaction between tidal zone and season had a highly significant effect on the morphological traits of *M. veneriformis*, suggesting that both tides and seasons are important factors influencing the morphological traits of *M. veneriformis*. In contrast, Johannesson et al. [39] analyzed the shell characteristics of *Littorina saxatilis* (Olivi, 1792) on the Spanish coast and found that the shell morphology varied significantly. Individuals in the intertidal zone were also significantly larger than those in the subtidal zone. Moreover, Watanabe et al. [40] found significant differences in the sharpness and thickness of the shells of *Ruditapes philippinarum* (A. Adams & Reeve, 1850) at different locations in the intertidal zone. Additionally, as the current speed increased, the thickness of the clam’s shell decreased and the shell became longer and flatter. In addition, Brake et al. [41] discovered that the shell width of Pacific oysters residing in turbulent intertidal currents was larger than those living in the intertidal zone with slow-flowing currents.

The body length–body mass relationship is usually used to describe the anisotropic growth of aquatic organisms [42]. It can be used to estimate individual body mass, understand the growth environment, and provide insights into the energy allocation of organisms during growth and reproduction [43]. In other studies, Xu et al. [44] found a correlation between the immune system of *Pinctada fucata martensii* (A. Gould, 1850) and its growth rate. Specifically, larger individuals of *P. martensii* had a weaker immune system compared with smaller individuals. This could be due to larger *P. martensii* individuals allocating more energy to growth and maintaining physiological homeostasis, whereas smaller individuals might allocate more energy to stress responses, potentially impacting their growth and development. Wen et al. [45] observed allometric growth in *Sinogastromyzon wui* (Fang, 1930) during its early developmental stages, with priority development of the head and body organs. This promotes predation and the evasion of natural enemies, supplies energy for the development of offspring and adaptation to the external environment, and enhances the survival rate. Additionally, Li et al. [32] found positive allometric growth in 10-month-old *Meretrix meretrix* (Linnaeus, 1758), isotropic growth in 15-month-old *Meretrix meretrix*, and negative allometric growth in 20-month-old *Meretrix meretrix*, which was related to suitable environmental conditions, such as temperature and salinity, abundant food, and few natural predators. In this study, *M. veneriformis* in both the middle and low tide zones exhibited positive allometric growth, suggesting a consistent energy allocation strategy. This may be due to the more consistent environment they experienced, suggesting that tidal differences in the northern Yellow River estuary habitat had a lesser impact on the growth of *M. veneriformis.*

### 4.2. Dietary Analysis

Currently, the feeding research methods for aquatic organisms include traditional methods (microscopy), biochemical methods (stable isotope analysis and fatty acid labeling), and molecular methods (high-throughput sequencing technology). Compared to other methods, high-throughput sequencing technology is more suitable for the identification of small, microscopic, and highly digestible organisms [46,47], and it is commonly used in animal feeding ecology studies on aquatic organism feeding habits [48].

In the nearshore waters of the Yellow River Delta, the dominant phytoplankton are Bacillariophyta, followed by Chlorophyta, Euglenozoa, and Chrysophyta [49]. Microscopic examination of the stomach contents of *M. veneriformis* showed that the composition was mainly Bacillariophyta [50]. The results of this study showed that the major phyla detected in the *M. veneriformis* stomach contents were Chrysophyta, Rhodophyta, and Chlorophyta. Bacillariophyta was likely not detected due to its preferential digestion and degradation within the digestive cycle cavity of *M. veneriformis*. We also concluded that these shellfish exhibit selective feeding behavior toward phytoplankton. In other studies, Zhang et al. [51] found that *Chlamys farreri* (K. H. Jones & Preston, 1904) preferentially consumed Pyrrophyta rather than Bacillariophyta. Jiang [52] observed that *Argopecten irradians* (Lamarck, 1819) ingested micro- and small-sized phytoplankton, particularly those with larger particle sizes. Moreover, *Synechococcus*, *Microchloropsis*, and *Chrysochromulina* are dominant phytoplankton genera in the pancreas of *Mercenaria mercenaria*, *Meretrix meretrix*, and *R. philippinarum*, respectively [53]. The specific reasons behind the preferences of *M. veneriformis* were not addressed in this study, but Bacillariophyta phytoplankton should be the focus of future research.

The NMDS analysis grouped samples from spring and summer into one category and those from fall and winter into another in the middle- and low-tide zones, which aligns with the temperature variations observed during the survey period. The ANOSIM analysis revealed that the majority of the inter-seasonal differences within the same tidal zone were significant (*p* < 0.05). Tidal differences in the northern Yellow River estuary habitat had a lower effect on the diet of *M. veneriformis*, and their diet was more affected by seasonal variations, with temperature showing significant seasonal variation. Temperature has been shown to significantly influence the phytoplankton community structure in the coastal waters of the Yellow River Delta [54,55]. Different phytoplankton species have different optimal temperatures for growth, and, thus, the structure of phytoplankton communities can change with temperature, leading to differences in the composition of the diet of shellfish.

The LEfSe analysis revealed that species within the phyla of green and red algae primarily occurred in the low-tide zone during spring and in the middle- and low-tide zones during winter. Gastropods have been found to have a larger biomass in the algal zone, likely because they prefer brown algae that are adapted to the substrate characteristics in this zone [56]. This adaptation enables gastropods to flourish under specific environmental conditions and to form morpho-functional groups that are well-suited to their habitats. Although filter-feeding shellfish cannot directly feed on macroalgae (e.g., *Bangia*), they can filter feed on their fragments. *Bangia* is rich in nutrients, such as proteins and unsaturated fatty acids, and it is usually found on intertidal rocks, which are exposed to environmental influences, such as sunlight, low temperatures, and rainfall, with the rise and fall of the tide [57]. The beaches along the northern Yellow River Delta are subject to strong tidal currents and coastal erosion [58], and *Bangia* is likely fragmented under these conditions, enabling filter-feeding by *M. veneriformis*. While our study offers valuable insights into the dietary characteristics of *M. veneriformis*, it is crucial to recognize the limitation posed by our relatively small sample size. With only four specimens per sample group, our findings may not fully encompass the variability within the population. Therefore, we recommend that future research employ larger sample sizes to corroborate our results and offer a more nuanced understanding of the species’ ecology

High-throughput sequencing, with its clear technical advantages, has been extensively applied in animal feeding research [28]. However, there are still some limitations, such as the inability to accurately quantify identification results [59], and the insufficiency of the current 23S rRNA database [60]. In this study, some of the OTUs could not be identified at the species level, highlighting the need for additional taxonomic information to enhance the database. Furthermore, high-throughput sequencing technology provides insights into the short-term feeding status of species and should be combined with other methods, such as stable isotope analysis, to gain a comprehensive understanding of long-term feeding habits. In the future, the taxonomic information coverage should be enhanced, and other feeding methods should be investigated to achieve a comprehensive understanding of *M. veneriformis* feeding in the intertidal zone of the Yellow River Delta.

## 5. Conclusions

The morphological characteristics of *M. veneriformis* in the northern intertidal zone of the Yellow River Delta are influenced by environmental factors. There are significant differences in morphological indicators between different intertidal zones, and there are also significant differences in the morphological characteristics of *M. veneriformis* in different seasons, indicating that tidal and seasonal differences have an impact on the morphological characteristics of *M. veneriformis*. High-throughput sequencing indicated that the predominant phyla in the stomach contents of *M. veneriformis* were Chrysophyta, Rhodophyta, and Chlorophyta. Differences in the feeding habits of *M. veneriformis* were more affected by seasonal variations than tidal differences, and seasonal temperature variations may be an important factor influencing the structure of phytoplankton communities, and ultimately the diet of *M. veneriformis*. This study provides foundational data that enhance our understanding of the feeding ecology of *M. veneriformis* and may support the development of conservation and management strategies. We acknowledge that further research is essential to address the understudied aspects of this species.

## Figures and Tables

**Table 1:** Seawater temperature of sampling sections in the Yellow River Estuary.

Groups	Temperature/°C	Groups	Temperature/°C
NLsp	24.45 ± 4.11 ^a^	NMsp	23.00 ± 3.98 ^a^
NLSu	26.08 ± 2.20 ^a^	NMSu	26.93 ± 3.18 ^a^
NLAu	14.66 ± 1.74 ^b^	NMAu	14.23 ± 2.46 ^b^
NLWi	14.52 ± 1.47 ^b^	NMWi	14.00 ± 2.03 ^b^

Different lowercase letters represented significant differences in water temperature between groups (*p* < 0.05).

**Table 2:** Allometric growth curve of *M. veneriformis.*

Group	Allometric Growth Equations
NMSp	BW = 2.245 × 10^−5^ × SL^3.703^
NLSp	BW = 1.268 × 10^−4^ × SL^3.179^
NMSu	BW = 2.253 × 10^−5^ × SL^3.702^
NLSu	BW = 8.378 × 10^−5^ × SL^3.312^
NMAu	BW = 1.998 × 10^−5^ × SL^3.738^
NLAu	BW = 7.643 × 10^−5^ × SL^3.321^
NMWi	BW = 2.274 × 10^−5^ × SL^3.670^
NLWi	BW = 5.541 × 10^−5^ × SL^3.422^

**Table 3:** Statistics of operational taxonomic units (OTUs) identification and classification status.

Groups	Sample Number	Phylum	Class	Order	Family	Genus	Species
NLSp	NLSp1	5	21	33	35	46	54
	NLSp2	4	17	25	25	32	37
	NLSp3	4	19	29	30	34	42
	NLSp4	5	20	33	34	48	57
NLSu	NLSu1	4	17	27	29	34	43
	NLSu2	3	13	18	18	21	27
	NLSu3	3	15	19	20	24	28
	NLSu4	4	18	25	26	28	33
NLAu	NLAu1	5	13	18	21	24	32
	NLAu2	3	13	19	19	22	32
	NLAu3	5	16	25	28	30	39
	NLAu4	4	14	23	23	26	34
NLWi	NLWi1	6	21	31	33	41	48
	NLWi2	4	10	11	13	15	18
	NLWi3	5	16	22	22	27	34
	NLWi4	5	17	31	35	40	53
NMSp	NMSp1	3	17	24	24	30	34
	NMSp2	3	17	22	20	25	31
	NMSp3	4	17	23	23	29	33
	NMSp4	4	17	24	27	32	36
NMSu	NMSu1	4	20	32	37	43	52
	NMSu2	5	22	38	42	53	72
	NMSu3	5	18	29	28	32	42
	NMSu4	5	21	37	41	48	59
NMAu	NMAu1	4	17	27	30	33	42
	NMAu2	5	15	20	23	29	35
	NMAu3	3	12	17	17	21	23
	NMAu4	5	13	17	17	19	21
NMWi	NMWi1	5	21	32	34	45	55
	NMWi2	6	24	36	44	54	72
	NMWi3	6	21	36	41	55	73
	NMWi4	6	20	29	34	44	51

## Data Availability

Data will be made available on request.
